# Comparing the neural correlates of affective and cognitive theory of mind using fMRI: Involvement of the basal ganglia in affective theory of mind

**DOI:** 10.2478/v10053-008-0129-6

**Published:** 2013-03-15

**Authors:** Maren E. Bodden, Dorothee Kübler, Susanne Knake, Katja Menzler, Johannes T. Heverhagen, Jens Sommer, Elke Kalbe, Sören Krach, Richard Dodel

**Affiliations:** 1 Clinic for Counseling and Psychotherapy, Institute of Psychology, Rheinische- Friedrich-Wilhelms-University Bonn, Germany; 2 Department of Neurology, Philipps-University Marburg, Germany; 3 Department of Radiology, Philipps-University Marburg, Germany; 4 Section of Brain Imaging, Department of Psychiatry and Psychotherapy, Philipps-University Marburg, Germany; 5 Institute for Gerontology, University of Vechta, Germany; 6 Department of Neurology, University of Cologne, Germany

**Keywords:** fMRI, affective and cognitive theory of mind, ToM, mentalizing, basal ganglia, simulation, social cognition

## Abstract

Theory of Mind (ToM) is the ability to infer other people’s mental states like intentions or desires. ToM can be differentiated into affective (i.e., recognizing the feelings of another person) and cognitive (i.e., inferring the mental state of the counterpart) subcomponents. Recently, subcortical structures such as the basal ganglia (BG) have also been ascribed to the multifaceted concept ToM and most BG disorders have been reported to elicit ToM deficits. In order to assess both the correlates of affective and cognitive ToM as well as involvement of the basal ganglia, 30 healthy participants underwent event-related fMRI scanning, neuropsychological testing, and filled in questionnaires concerning different aspects of ToM and empathy. Directly contrasting affective (aff) as well as cognitive (cog) ToM to the control (phy) condition, activation was found in classical ToM regions, namely parts of the temporal lobe including the superior temporal sulcus, the supplementary motor area, and parietal structures in the right hemisphere. The contrast aff > phy yielded additional activation in the orbitofrontal cortex on the right and the cingulate cortex, the precentral and inferior frontal gyrus and the cerebellum on the left. The right BG were recruited in this contrast as well. The direct contrast aff > cog showed activation in the temporoparietal junction and the cingulate cortex on the right as well as in the left supplementary motor area. The reverse contrast cog > aff however did not yield any significant clusters. In summary, affective and cognitive ToM partly share neural correlates but can also be differentiated anatomically. Furthermore, the BG are involved in affective ToM and thus their contribution is discussed as possibly providing a motor component of simulation processes, particularly in affective ToM.

## Introduction

The human ability to infer other people’s mental states such as intentions, emotions, or desires, namely Theory of Mind (ToM; cf. Premack & Woodruff, 1978), provides an essential basis for successful social interaction by enabling the prediction of other’s most probable future acts (Frith & Frith, 1999). The ability to appreciate the emotional states of a counterpart deepens social relationships. Therefore, the complex neuropsychological construct ToM is gathering vast interest in recent neuroscientific research (e.g., Adolphs, 2003). Successfully applying ToM in social interactions facilitates human relationships and attachment while impairment of ToM abilities have been described in various psychiatric diseases. Recently, ToM deficits have also been reported in neurological disorders and have been linked to the basal ganglia (BG; see Alegre et al., 2010; Bodden, Dodel, & Kalbe, 2010). In particular, ToM dysfunctions have been reported in patients suffering from neurodegenerative disorders such as Parkinson’s disease (Bodden, Mollenhauer, et al., 2010; Péron et al., 2009) and Huntington’s disease (Snowden et al., 2003). Another functional system of social cognition that interacts closely with the ToM network is the human mirror neuron system. Alegre and colleagues (2010), who examined ToM abilities of patients suffering from Parkinson’s disease in an EEG study, have proposed the involvement of the BG in this system as well.

A widespread network entailing the sulcus temporalis superior, the temporoparietal junction, the temporal poles, the ventromedial prefrontal, and the orbitofrontal cortex amongst other regions was suggested to form the neuroanatomical basis of ToM (Amodio & Frith, 2006; Carrington & Bailey, 2009; Saxe, Carey, & Kanwisher, 2004). To which extent the amygdala is contributing to the network during ToM processing is currently under discussion (Adolphs, 2010). The structures mentioned above are considered as the core regions involved in ToM abilities (Carrington & Bailey, 2009). The finding that ToM dysfunctions are common in BG related neurological disorders leads to question a possible involvement of the BG in ToM. Altogether, the results of recent functional imaging studies examining the neural correlates of ToM are partly heterogeneous. To some extent this may be due to different ToM paradigm types such as cartoons (Gallagher et al., 2000), written scenarios (Happé, Brownell, & Winner, 1999), and other animated figures that have been used (Castelli, Happé, Frith, & Frith, 2000). Another source of heterogeneity of the results are the different concepts examined (ToM, empathy, etc.) as well as their operationalization. The ability to recognize or infer others’ feelings or mental states (ToM) does not mandatorily entail empathy, defined as the ability to share others’ feelings (Singer & Lamm, 2009). Finally, conditions examining different subcomponents of the ToM concept were not always kept as comparable as possible.

The multifaceted construct ToM can be sub-divided into affective (i.e., recognizing the feelings of another person) and cognitive (i.e., inferring the mental states of the counterpart, his/her desires, beliefs, or intentions) subcomponents (Eslinger, 1998; Shamay-Tsoory, Aharon-Peretz, & Levkovitz, 2007), each of which can be affected individually or in combination (Harari, Shamay-Tsoory, Ravid, & Levkovitz, 2010; Péron et al., 2009). Various terms and definitions have been used in the literature to describe these subcomponents (Kalbe et al., 2007), including *emotional* versus *cognitive perspective taking* (Hynes, Baird, & Grafton, 2006), *empathy* versus *ToM* (Völlm et al., 2006) or *affective* versus *cognitive ToM* (Shamay-Tsoory et al., 2007). If subcomponents were specified and examined, mostly an affective as well as a cognitive component were differentiated. At present it still remains indistinguishable whether affective and cognitive ToM abilities are to be differentiated due to the contents of the ToM process (belief or intention vs. emotional state; see Shamay-Tsoory & Aharon-Peretz, 2007) or due to different underlying processes (simulating or rather mirroring vs. mental perspective taking in the sense of inferring the mental states of others based on a theory of the mental world; see Adolphs, 1999; Van Overwalle & Baetens, 2009).

The systematic investigation of affective and cognitive ToM has only recently been initiated and thus only a few functional imaging studies have compared both subcomponents. Different activation patterns referring to these subcomponents have been described (Hynes et al., 2006; Völlm et al., 2006) and it is suggested that the affective and cognitive ToM abilities recruit overlapping but partially distinct neural networks (Völlm et al., 2006). While affective ToM abilities seem to be mediated by the ventromedial prefrontal cortex (Shamay-Tsoory & Aharon-Peretz, 2007) and orbitofrontal cortex (Hynes et al., 2006), cognitive ToM abilities have been associated especially with dorsolateral prefrontal regions (Eslinger, 1998; Kalbe et al., 2010; Montag, Schubert, Heinz, & Gallinat, 2008). In order to highlight this difference in the neural correlates of affective and cognitive ToM, both subcomponents should be investigated using highly comparable stimulus material.

In the present study, we investigated whether the neural activation patterns of affective and cognitive ToM can be distinguished using a paradigm with highly comparable ToM conditions (adapted from Shamay-Tsoory et al., 2007; German version by Kalbe et al., 2010). Furthermore, we examined the involvement of the BG in ToM and more precisely their contribution to affective and cognitive ToM. Therefore, in addition, behavioural data of several ToM questionnaires applied and the behavioural data of the Yoni task (Shamay-Tsoory et al., 2007) derived from the scanning session were related to a possible activation within the BG during the ToM task.

## Methods

### Subjects

Thirty-five right-handed (Edinburgh Handedness Inventory score > 80; cf. Oldfield, 1971) native German speaking participants were scanned. Of these 35 participants, five were excluded from the study due to reasons including technical problems during data acquisition (one participant), Beck Depression Inventory-II (BDI-II) scores of clinical relevance (> 14; two participants), or head movement during the scanning procedure (two participants). Of the remaining 30 participants (15 women, 15 men; *M*_age_ = 25.3, *SD*_age_ = 2.5 years, age range from 20 to 30; years of education: *M* = 13.9, *SD* = 2.2 years) none had a history of neurological or psychiatric disease and nobody used psychotropic drugs. This study was approved by the local ethics committee of the Philipps-University Marburg, and all participants gave written informed consent before enrolment.

### Neuropsychological tests and questionnaires

For the assessment of verbal learning and memory, the Rey Auditory-Verbal Learning Test Trial (RAVLT; Schmidt, 1996) was conducted. Working memory was evaluated using the digit span forward and backward from the revised version of the Wechsler Memory Scale (WMS-R; Härting et al., 2000). Further, the Corsi blockspan test (Härting et al., 2000) was administered. Executive functions were assessed applying 1-min lexical and semantic verbal fluency tasks with the letters F, A, and S, the category “groceries” (Aschenbrenner, Tucha, & Lange, 2000), and the Trail Making Test (TMT; Tombaugh, 2004). Furthermore, reasoning was measured by the Subtest 4 of the German intelligence test battery *Leistungsprüfsystem* (LPS 4; Horn, 1983), and crystallized intelligence was measured by the German vocabulary test.

The BDI-II (Hautzinger, Keller, & Kühner, 2006) was used as to screen for symptoms of depression. Furthermore, all participants filled in a German version of the Interpersonal Reactivity Index (IRI; Paulus, 2006) to measure empathy according to the four subscales (*perspective taking, fantasy, empathic concern, and personal distress*), as well as the Empathy-Scale (E-Scale; Leibetseder, Laireiter, Riepler, & Köller, 2001) which includes the subscales *cognitive sensitivity, emotional sensitivity, cognitive concern, and emotional concern*. Some subscales of these questionnaires measure different aspects of the multifaceted construct ToM. Additionally, the Reading the Mind in the Eyes Test (RMET) was administered (Baron-Cohen, Wheelwright, Hill, Raste, & Plumb, 2001). The RMET is a well known ToM task in which participants have to choose one of four words that they believe best describes the mental state of a character. In the task, only photographs of eye regions are presented.

### Statistical analysis

For each neuropsychological test, the mean score of the group was calculated and compared to norm data for the appropriate age. All neuropsychological test scores were correlated with the mentalizing scores (i.e., the IRI and E-Scale and the RMET as well as behavioural data of the Yoni task). Instead of Bonferroni correction for multiple comparisons, a more conservative alpha-level (*p* = .01) was chosen for this particular analysis.

### fMRI stimulus material

**Figure 1.**
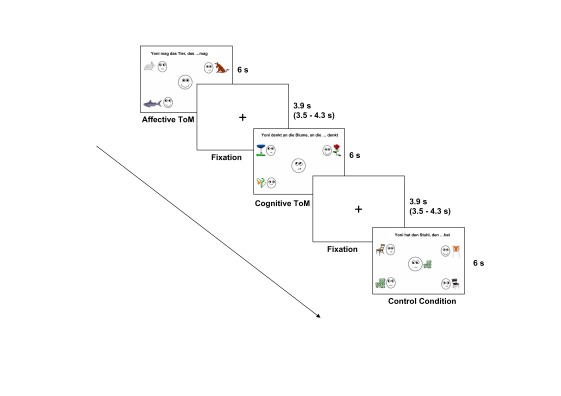
Design of the Yoni task. ToM = Theory of Mind.

For the present study, the Yoni task, a paradigm introduced by Shamay-Tsoory and colleagues, was adapted for the fMRI environment (Shamay-Tsoory et al., 2007). In the stimulus material, the face of the main character named “Yoni” was located in the centre of the screen. Four other coloured pictures in the corners showed faces, each in combination with one object of a semantic category (e.g., flowers, toys, fruits; see Figure 1). Three conditions consisting of 20 items each were distinguished: affective ToM (aff), cognitive ToM (cog), and a control condition (phy). Statements written on the upper margin of the screen which should be completed by the participants were as follows: “Yoni likes the fruit that … likes.” (example for the aff condition); “Yoni is thinking of the flower that … is thinking of.” (example for the cog condition); and “Yoni has the toy that … has.” (example for the phy condition). All three conditions were kept almost identical and only differed in the shape of the mouth of Yoni as well as the verb of the sentence. Whereas mentalizing was needed for both the affective and the cognitive condition, processing of control items required only an analysis of physical attributes. Every item had only one correct answer in which both the facial expression and the eye gaze reflected what was said in the sentence (ambiguity of the task had been checked in a behavioural study). Facial expressions and eye gaze direction of the four faces in the corners were systematically balanced, that is, in half of the items Yoni’s eye gaze was straight, in the other half Yoni’s eye gaze was towards the direction of the correct choice, and in half of the items two of the small faces had the same facial expression as Yoni in order to avoid simple face matching.^1^ At task participants had to choose one out of four possibilities which best completed the sentence. They indicated the corner of the screen where they think the answer was located by pressing the button corresponding to it. Participants had been trained on the use of the button box before the start of the scan inside the scanner. Summing up, the solution of the task is based on the integration of verbal cues (sentence), facial expressions (shape of mouth), and eye gaze.

### fMRI procedure

An event-related design including the three conditions described above and a fixation cross in between the items serving as low level baseline was applied. Each of the 60 items was displayed for 6 s. The fixation cross was jittered from 3.5 to 4.3 s (*M* = 3.9 s; cf. Figure 1). Presentation of stimuli was controlled using the Presentation 11.0 software package (Neurobehavioral Systems, Albany, CA, USA; see http://www.neurobs.com/).

In a test run within the scanner, participants were trained to use four different response buttons to indicate their choices. After having completed the training task successfully, fMRI scanning was started, and all responses were recorded for subsequent data analyses. The images were rear-projected on a screen (that was located 200 cm from the head coil) and were visible via a mirror that was attached to it. Participants laid in a supine position and head movement was limited by foam padding within the head coil. For each participant, a series of 200 EPI-scans lasting 9 min 54 s in total was acquired. The initial five images were excluded from further analysis in order to remove the influence of T1 stabilization effects.

### fMRI data acquisition and analysis

The study was conducted on a 1.5 T MRI (magnetic resonance imaging) Scanner (Siemens Magnetom Sonata) with a conventional head coil to acquire whole brain MRI data. A standard BOLD-sensitive EPI-sequence was used to acquire functional images (TE: 50 ms; TR: 3,000 ms; slice thickness: 3.5 mm with a 10% gap between the slices [0.35 mm]; flip angle: 90°; voxel size 3.5 × 3.5 × 4.2 mm^3^, FoV: 225 mm; matrix: 64 × 64). After the functional scanning procedure, two sagitally oriented T1-weighted volumes were acquired for coregistration.

SPM8 (www.fil.ion.ucl.ac.uk/spm) standard routines and templates were used for analysis of fMRI data. The functional images were realigned, normalized (resulting voxel size 2 × 2 × 2 mm^3^), smoothed (8 mm isotropic Gaussian filter), and high-pass filtered (cut off period = 128 s). Supplementary, temporal and dispersion derivatives were included in the analysis. Statistical analysis was performed in a two-level, mixed-effects procedure. At the first level, BOLD responses for the conditions aff, cog, and phy were modeled by a stick function convolved with the canonical hemodynamic response function employed by SPM. Parameter estimates (ß-) and *t*-statistic images were calculated for each subject.

For second level analysis, the ß-contrasts of the affective, cognitive, and control condition obtained from the first level relative to the baseline were entered into a full factorial design. Initially, group activation maps related to each condition as well as the deactivation were calculated. Monte Carlo simulation (S. Slotnick, Boston College, *n* = 1,000) of the brain volume indicates that using a statistical criterion of 46 or more contiguous voxels at a voxelwise threshold of *p* < .001 provides a brain-wise alpha level of *p* < .05, corrected for multiple comparisons. Activation maps for the contrasts of interest (aff > phy, cog > phy, aff > cog, and cog > aff) were identied. The anatomical localization of activated brain regions was assessed by the SPM anatomy toolbox (Eickhoff et al., 2005).

To analyze the activation within the BG, beta values from the anatomically defined region of interest (ROI) of the BG were derived for all three conditions as well. For the anatomical specification of the BG we used the SPM anatomy toolbox (Eickhoff et al., 2005). Therefore, we included the regions of the caudate nucleus, the Globus pallidus and the Putamen. Beta values were driven from all three conditions of the Yoni task from the first level data sets (i.e., from the individual scans of each participant). The extracted data was then correlated with the data from the questionnaires as well as with the behavioural data.

## Results

### Behavioural results

None of the participants showed cognitive deficits in the neuropsychological tests applied. The results are presented in Appendix A (Table A1).

**Figure 2.**
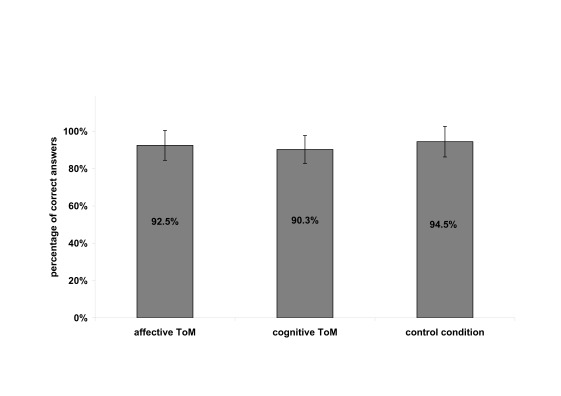
Behavioural data of the Yoni scales.

On average, participants solved 68.3%, *SD* = 9.6, of the RMET items correctly. In the Yoni task, participants solved 92.5%, *SD* = 8.0, of the items in the affective condition. In the cog condition, 90.3%, *SD* = 7.5, of the items were answered correctly (94.5%, *SD* = 8.2, in the phy condition; see Figure 2). Neither the comparison of the affective to the control condition (*p* = .443) nor of the cognitive to the control condition (*p* = .122) nor a comparison of affective and cognitive condition (*p* = .470) showed any significant difference on the behavioral level.

Results from the correlations between neuropsychological data and behavioural ToM data scores are displayed in Table A1. Only a few significant correlations were found and included: the delayed recall of the RAVLT (A7) with the control scale of the Yoni task (*p* = .006), the lexical verbal fluency (*F*, *A*, *S*) with the control scale of the Yoni task (*p* = .002) as well as with the RMET (*p* = .003).

#### Questionnaires

In the IRI, participants had an average value of 26.3, *SD* = 3.6, in the subscale *perspective taking*, 24.7, *SD* = 4.3, in *fantasy*, 25.3, *SD* = 3.9, in *empathic concern*, and 16.5, *SD* = 3.7, in *personal distress*. In the subscales of the E-Scale, they scored on average 2.1, *SD* = 0.9, in *cognitive sensitivity*, 2.5, *SD* = 0.8, in *emotional sensitivity*, 1.9, *SD* = 0.8, in *cognitive concern*, and 2.4, *SD* = 0.7, in *emotional concern*.

There were no correlations between any neuropsychological data and results from the questionnaires. Neither were any correlations found between the behavioural data of the Yoni task and the scales from the questionnaires applied. Only age correlated with the subscale *fantasy* from the IRI (*p* = .004; cf. Table A1).

### fMRI results

#### Affective ToM

**Figure 3.**
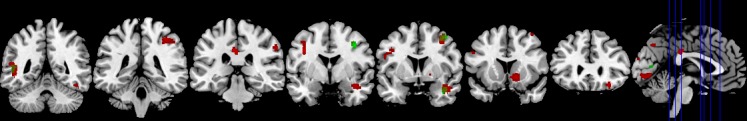
Significant activation clusters of the affective (red) and cognitive (green) over control condition. *T* = 3,19; *p* < .05; cluster threshold = 46 voxels. The blue lines in the sagittal view indicate the coronar slice levels from occipital to frontal.

Affective ToM contrasted to control (aff > phy) yielded significant activation of the right inferior temporal gyrus and the right superior temporal sulcus (STS, BA 21/22). The latter cluster was partly extending into the amygdala. Additionally, the orbitofrontal cortex on the right and the middle cingulate cortex on the left, as well as the supplementary motor area (SMA, BA 6) on the right hemisphere were strongly implicated in affective ToM. The left precentral and inferior frontal gyrus (BA 44/45) and parts of the right inferior parietal cortex and the right precuneus extending to the other hemisphere were recruited as well. Subcortically, the caudate nucleus and the pallidum, both lateralized to the right hemisphere, were found activated (see Figure 3). Finally, a cluster in the left cerebellum was activated (see Table A2, clusters restricted to the visual cortex are not reported).

#### Cognitive ToM

The processing of the cognitive subcomponent of ToM contrasted to control (cog > phy) elicited activation in the right SMA (BA 6) and the right STS that extended into the amygdala, the right parietal lobule, and the left middle temporal gyrus including the temporo-parietal junction (TPJ).

**Figure 4.**
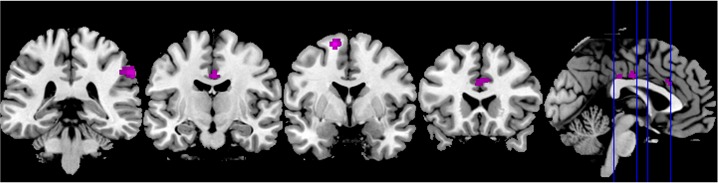
Significant activation clusters of the contrast of the affective over cognitive condition. *T* = 3,19; *p* < .05; cluster threshold = 46 voxels. The blue lines in the sagittal view indicate the coronar slice levels from occipital to frontal.

The direct contrast of the affective with the cognitive subcomponent of ToM (aff > cog) revealed significant activation of the right inferior parietal cortex extending into the TPJ. In addition, the left SMA (BA 6), the right anterior cingulate cortex, and the middle cingulate cortex bilaterally were more strongly implicated during affective as opposed to cognitive ToM. Furthermore, this contrast yielded activation in the left precentral gyrus extending into the somatosensory cortices (Figure 4).

The reversed contrast (cog > aff) did not yield any significant activation clusters.

### Correlations between BG activation and questionnaires

As described above, activation was found in the BG when participants processed the items of the affective ToM condition. In an analysis of the anatomically defined ROI of the BG, a correlation was found between the levels of activation (beta values) in the affective Yoni condition and the subscales *perspective taking* (*p* = .033) and *distress* (*p* = .013) of the IRI (cf. Table A1). There were no further correlations to other subscales, the RMET or the Yoni scales. Moreover, activation during ToM processing did not correlate with any data of the neuropsychological test scores.

## Discussion

Social cognition processes consist of various subcomponents. Thus, the concept ToM has been referred to as an “umbrella term” (Hynes et al., 2006) comprising different processes. We conducted this study with the aim of finding out more about the neural correlates of ToM and its different subcomponents. There are two main findings of this study: First, affective and cognitive ToM components can be differentiated on a neural level. This is in line with the results of previous examinations (Hynes et al., 2006; Völlm et al., 2006). Second, the BG are involved in affective, but not in cognitive ToM processing.

This investigation differs from previous research concerning the differentiation of affective and cognitive ToM in the paradigm used for this study. In contrast to other examinations (Hynes et al., 2006; Völlm et al., 2006), the Yoni task entails three highly comparable conditions which only differ concerning the verb used (*like* vs. *think* vs. *have*) and Yoni’s mouth. The content of the visual stimuli as well as the conceptual formulation for the participants are almost identical across all conditions. Thus, different activation patterns found seem to be evoked by slight differences of content between the three conditions. As a limitation to our study design the influence of the different verbs used in the three different conditions on the activation patterns found cannot be exactly defined.

Considering the items of the Yoni task, one may argue that this paradigm is hardly able to measure sophisticated human abilities such as ToM. Nevertheless, it has been applied successfully in behavioral (Shamay-Tsoory, 2008), TMS- (Kalbe et al., 2010), and lesion studies (Shamay-Tsoory & Aharon-Peretz, 2007; Shamay-Tsoory et al., 2007) for the differentiation of affective and cognitive ToM. Indeed, the advantage of the Yoni paradigm lies in its simplicity. What is necessary to solve the Yoni task, can be defined precisely as the ability to integrate different cues (i.e., to say, verbal cues, facial expressions, and eye gaze), all considered aspects of the sophisticated social mentalizing process (Frith & Frith, 2006).

In general, the results are in line with previous research (e.g., Adolphs, 2002; Hynes et al., 2006). Involvement of the parietal cortex during ToM processing was also reported by Rizzolatti and Sinigaglia (2010), who specified the fronto-parietal mirror circuit. Summing up, the results of the present study corroborate the suggestion that ToM in general recruits a network of brain structures, irrespective of the differentiation between its affective and cognitive subcomponents (Völlm et al., 2006). Affective and cognitive ToM share neural correlates but can also be differentiated on an anatomic basis. These results also suggest that affective ToM recruits additional regions compared to cognitive ToM, especially medial parts of the frontal cortex as has been found in previous research (Hynes et al., 2006). Thus, this study supports the hypothesis that ToM serves as an “umbrella term” (Hynes et al., 2006), that is, a concept entailing different subcomponents.

Recently, a distinction of the level of processing has been proposed by Van Overwalle and Baetens (2009) who differentiate between a mirror and a mentalizing system. The mirror system, consisting of the anterior intraparietal sulcus and the premotor cortex, is engaged when perceiving biological motion and grasping the underlying intentions of the observed movement. The mentalizing system, comprising the temporoparietal junction, the medial prefrontal cortex, and the precuneus, provides a basis for a more abstract inference of goals or intentions (when no action of body parts is observable and when intentions need to be inferred from abstract cues such as eye gaze, semantic information, facial expression, or knowledge about the situation).

Interestingly, activation was found in structures of the BG, namely, the caudate nucleus as well as the pallidum on the right hemisphere in the affective condition contrasted to the control condition. The direct contrast aff > cog did not show additional activation within the BG. One possible explanation for this is that the cognitive ToM condition yields more activation than the control condition but not as much activation as the affective condition does. The subcomponents cannot be understood as completely distinct conditions, thus, affective and cognitive ToM might overlap. Unfortunately, the event-related design applied impedes a definitive conclusion. Beta values derived from an anatomical defined ROI of the basal ganglia (Eickhoff et al., 2005) from the affective Yoni condition correlated with two subscales of the IRI, namely with *perspective taking* as well as with *distress*. Taking into account that the IRI contains four subscales and thus adapting the alpha level, one may argue that the correlation between beta values and *perspective taking* (*p* = .033) should be considered as a statistical trend. The subscale perspective taking was conceptualized as to assess “the tendency to spontaneously adopt the psychological point of the view of others” (Davis, 1983, pp.113-114), which may be reminiscent of cognitive aspects of the ToM concept, whereas Shamay-Tsoory and colleagues (2007) reported a correlation between this subscale and the affective condition of the Yoni task similar to our findings. However, affective ToM seems to be processed more spontaneously than cognitive ToM. Thus, the actual ToM subcomponent assessed by this subscale might be viewed as controversial.

The subscale *personal distress* is thought to measure typical emotional reactions of the recipient, in particular “personal anxiety and unease in tense interpersonal settings” (Davis, 1983, p. 114). The questions of this scale involve aspects of emotional regulation as well as witnessing of another person being in an emergency. This can be conceptualized as an aspect of affective ToM as, in particular, it automatically mirrors other people’s emotions.

ToM dysfunctions are common in various BG disorders (e.g. in Parkinson’s disease) and have been associated with frontostriatal dysfunctions (Bodden, Dodel, & Kalbe, 2010; Roca et al., 2010) as well as with atypical Parkinson’s disease syndromes (O’Keeffe et al., 2007) and with Huntington’s disease (Snowden et al., 2003). Additionally, involvement of the BG in the human mirror neuron system was recently discussed by Alegre and colleagues (2010) further illustrating their impact in various cognitive abilities. It has been proposed that perceiving others’ emotional states triggers mirroring this emotion in the recipient and that the BG are involved in this connection (Adolphs, 2002). Thus, further insight in BG involvement in social cognition might be obtained by investigating patients with corresponding disorders in functional imaging studies.

It may be speculated that the BG are involved in simulation in terms of providing a motor component for this process. Alternatively, the BG might be involved in affective ToM due to their impact on emotion recognition and facial expression decoding (Assogna, Pontieri, Caltagirone, & Spalletta, 2008). Following the suggestions made by Van Overwalle and Baetens (2009), the BG may be involved in mirroring other peoples’ mental states by simulation in contrast to more explicit mentalizing which requires higher level cognitive operations. Mirroring or simulating mental states of others is mostly thought to be associated with the affective ToM subcomponent (e.g., Kalbe et al., 2007). The interpretation of our findings in the way depicted above provides a coherent conclusion which is in line with the studies mentioned. Nevertheless this remains quite speculative and requires further research. Only a task requiring both a definitive distinction on the content and the process level could clarify this issue. Furthermore, mentalizing and mirroring strategies, although different processes, work in close conjunction with each other. People refer to these two processes when trying to grasp the mental states of others although the extent to which processes are used can vary among individuals. Another possible contribution of the BG to ToM is their involvement in cognitive flexibility (Niendam et al., 2012). The ability to adopt the mental perspective of another person requires at least to a certain extent cognitive flexibility. However, this hypothesis could not explain a differentiation of activity between the affective and cognitive condition. Further research is needed to specify the differentiation as well as the relationships between both subcomponents. In order to look at circuitry involving the BG as part of the ToM network, functional imaging studies involving patients with BG disorders might be fruitful to elucidate the hypothesized distinction on a process level.

## Footnotes

1 The perfect balance of the possible gaze direction respectively its congruence with the correct answer was compromised when incorrectly answered items were excluded from the analysis. Thus, errors in the different conditions were counted, and no systematic errors were found. Regarding the cognitive items, we cannot find a difference. In the affective condition, there were more errors made when the face in the middle gazes straight forward. In our opinion, this finding does not influence the major results because the straight forward condition is not more difficult and does not produce more errors than the comparable cognitive condition, whereas the condition in which the gaze direction is congruent with the right answer is easy to solve. Furthermore, it is impossible to discern between types of errors, for example, errors due to presses of the wrong button or due to choosing the wrong item. Unfortunately, this possible criticism of the study cannot be eliminated. Participants were trained in using the answer box within the scanner, but we think it was not possible to eliminate completely any misses (while pressing the buttons). Nevertheless, we think, this does not influence the major results, because we do not primarily analyse the behavioural data. We were focusing on the processes on a neural level.

## Author Note

M. E. Bodden and D. Kübler contributed equally.

M. E. Bodden received funding by the von Behring Röntgen Stiftung. The authors report no conflicts of interest: The funding did not have any influence on study design, collection, analysis, and interpretation of data nor in other aspects regarding this manuscript.

We thank Simone Shamay-Tsoory for kindly providing the Yoni paradigm.

## Appendix A

**Table A1.** Neuropsychological Test Data of the Participants and Correlations Between Neuropsychological Data and Theory of Mind Scores

				*p* value correlations^a^			
	Participants(*N* = 30)	Min-max(range)	Norm data	IRI			
				*Fantasy*	*Empathy*	*Perspective taking*	*Distress*
Female, *n* (%)	15 (50%)	-	-				
Age, y	25.3 ± 2.5	20-30	-	.505*	.049	.059	-.412
				.004	.797	.755	-.024
Education, y	13.9 ± 2.16	10-18	.	.083	.331	.231	-.136
				.662	.074	.218	.475
Memory							
Rey auditory verbal learning test, immediate recall (total of 5 trials)	63.0 ± 5.49	52-73	56.7 (7.3)	.186	.180	.060	.162
				.326	.341	.754	.393
Rey auditory verbal learning test, inference condition	9.3 ± 2.51	5-13	6.7 (2.0)	.352	.151	.086	.314
				.056	.424	.653	.091
Rey auditory verbal learning test, delayed recall (after inference condition)	13.63 ± 1.65	9-15	11.5 (2.3)	.036	-.040	-.111	.180
				.849	.835	.558	.341
Rey auditory verbal learning test, delayed recall (after 30 min)	13.43 ± 2.11	6-15	11.3 (2.5)	.140	.108	-.025	.297
				.460	.569	.896	.111
Digit span forward(WMS-R) points (items)	8.83 ± 1.64	(6-12)	PR: 68-48	-.162	-.061	.201	-.004
	(7.07 ± 0.94)	(5-9)		.392	.747	.286	.984
Digit span backward(WMS-R) points (items)	8.17 ± 2.07	(5-13)	PR: 80-53	.196	-.050	.078	.249
	(5.83 ± 1.34)	(4-8)		.300	.792	.684	.185
Block span forward points (items)	9.33 ± 1.71	(6-14)	PR: 65-50	-.003	-.158	-.269	.170
	(6.37 ± 1.23)	(4-8)	PR: 65-50	.987	.404	.151	.370
Block span backward points (items)	9.67 ± 1.27	(7-12)	PR: 78-75	-.009	-.068	-.306	.012
	(6.3 ± 0.92)	(4-7)	PR: 78-75	.964	.722	.100	.949
Executive functioning and reasoning							
Lexical verbal fluency (letters *F*, *A*, *S*)	48.0 ± 9.31	32-60	PR: 50	.289	-.052	.026	.079
				.121	.786	.892	.676
Semantic verbal fluency (groceries)	30.53 ± 6.18	21-44	PR: 75	.275	.100	.003	.013
				.142	.598	.989	.947
TMT B / A, time B / time A	2.16 ± 0.51	(1.3-3.3)	Percentile 40	-.146	-.275	.147	.133
				.442	.141	.437	.483
Reasoning (LPS)	34.87 ± 2.9	27-41	C: 9	.168	-.014	.338	-.043
				.374	.942	.068	.822
Verbal intelligence test ("Wortschatz Test")	34.87 ± 2.9	27-41	.	-.035	-.076	.426	-.138
				.856	.690	.019	.468
Non-cognitive domains							
BDI-II	1.83 ±2.51	0-10	Cut off for clinical relevance ≥ 14	.144	-.012	-.356	.289
				.447	.950	.053	.121

**Table A1. (Continued):** Neuropsychological Test Data of the Participants and Correlations Between Neuropsychological Data and Theory of Mind Scores

	Participants(*N* = 30)	Min-max(range)	Norm data	*p* value correlations^a^							
Empathy Scale				Yoni			RMET
E_CS	E_ES	E_EC	E_CC	aff	cog	phy
Female, *n* (%)	15 (50%)	-	-								
Age, y	25.3 ± 2.5	20-30	-	-.418	-.363	-.258	-.3397	-.234	-.100	-.298	.117
				.022	.049	.168	.067	.230	.611	.124	.539
Education, y	13.9 ± 2.16	10-18	.	-.095	.075	.104	-.057	-.089	.097	.162	-.061
Education, y	13.9 ± 2.16	10-18	.	.617	.692	.586	.763	.653	.624	.411	.749
Memory											
Rey auditory verbal learning test, immediate recall (total of 5 trials)	63.0 ± 5.49	52-73	56.7 (7.3)	.171	.141	.291	.342	-.182	.122	.357	.250
				.366	.458	.119	.065	.354	.535	.062	.183
Rey auditory verbal learning test, inference condition	9.3 ± 2.51	5-13	6.7 (2.0)	.107	.188	.169	.188	-.066	-.015	.174	.308
				.574	.319	.372	.319	.740	.940	.375	.097
Rey auditory verbal learning test, delayed recall (after inference condition)	13.63 ± 1.65	9-15	11.5 (2.3)	.057	-.071	.073	.218	-.044	.224	.430	.169
			)	.767	.710	.703	.248	.826	.253	.023	.372
Rey auditory verbal learning test, delayed recall (after 30 min)	13.43 ± 2.11	6-15	11.3 (2.5)	.224	.112	.224	.364	-.136	.089	.504*	.195
			)	.234	.554	.234	.048	.491	.651	.006	.302
Digit span forward(WMS-R) points (items)	8.83 ± 1.64	(6-12)	PR: 68-48	-.113	-.027	.045	.006	.055	-.0589	-.072	-.163
	(7.07 ± 0.94)	(5-9)		.553	.887	.813	.974	.782	.769	.714	.388
Digit span backward(WMS-R) points (items)	8.17 ± 2.07	(5-13)	PR: 80-53	.074	.200	.106	.003	-.017	-.044	.283	.029
	(5.83 ± 1.34)	(4-8)		.696	.290	.577	.985	.933	.823	.144	.877
Block span forward points (items)	9.33 ± 1.71	(6-14)	PR: 65-50	-.136	-.142	-.095	-.088	.179	.270	.303	.285
	(6.37 ± 1.23)	(4-8)		.474	.453	.618	.645	.363	.165	.117	.127
Block span backwardpoints (items)	9.67 ± 1.27	(7-12)	PR: 78-75	-.175	.004	.088	.057	.330	.052	.131	-.166
	(6.3 ± 0.92)	(4-7)		.356	.984	.642	.766	.086	.795	.506	.380
Executive functioning and reasoning											
Lexical verbal fluency(letters *F*, *A*, *S*)	48.0 ± 9.31	32-60	PR: 50	.180	.232	.060	.190	-.187	.110	.564*	.519*
				.342	.217	.753	.314	.341	.578	.002	.003
Semantic verbal fluency (groceries)	30.53 ± 6.18	21-44	PR: 75	.330	.267	.206	.282	.174	.445	.228	.165
				.075	.154	.275	.132	.377	.018	.243	.385
TMT B / A, time B / time A	2.16 ± 0.51	(1.3-3.3)	Percentile 40	-.204	.093	-.151	-.075	.128	-.073	-.345	-.169
				.280	.624	.425	.695	.518	.710	.072	.373
Reasoning (LPS)	34.87 ± 2.9	27-41	C: 9	.231	.398	.175	.202	.225	-.017	.237	-.008
				.219	.029	.355	.284	.251	.933	.225	.965
Verbal intelligence test ("Wortschatz Test")	34.87 ± 2.9	27-41	.	.033	.169	-.056	.104	-.1063	-.081	-.0973	.137
				.864	.373	.768	.584	.593	.682	.623	.469
Non-cognitive domains											
BDI-II	1.83 ±2.51	0-10	Cut off for clinical relevance ≥ 14	.045	-.188	.081	-.048	.139	.043	.036	.177
				.814	.320	.671	.802	.481	.823	.855	.367

*Note*. Data shown as mean +/- standard deviation and minimum to maximum score. Norm data: the norm for the mean per group is provided. aff = affective theory of mind condition. BDI-II = Beck Depression Inventory-II. cog = cognitive theory of mind condition. IRI = Interpersonal Reactivity Index. LPS = Leistungsprüfsystem. phy = control condition. TMT = Trail Making Test. WMS-R = Wechsler Memory Scale – Revised. ^a^Alpha level was set at *p* = .01 instead of Bonferroni correction. *Correlation significant at α = .01 (two-tailed).

**Table A2.** Localization of Significant Clusters

		MNI-Coordinates						
	Anatomical region	Hemisphere	BA	x	y	z	*t*-value	Cluster size
cog > phy	Middle frontal gyrus as part of SMA	R	6	36	4	52	4.51	125
	STS extending to amygdala	R	21/22	40	2	-26	4.20	60
	Superior parietal lobule	R	7	24	-64	50	3.89	58
	Middle temporal gyrus extending to TPJ	L	21	-48	-50	8	3.80	46
aff > phy	Inferior temporal gyrus	R	37	46	-54	-16	4.38	294
	Precentral and inferior frontal gyrus	L	44/45	-38	-4	34	4.44	238
	STS extending to amygdala	R	21/22	42	4	-24	4.54	190
	Middle frontal gyrus as part of SMA	R	6	36	4	52	5.30	170
	Caudate and pallidum	R		14	16	-8	4.23	159
	Inferior parietal lobule	R	2	38	-40	50	4.09	115
	Precuneus	R/L	7	8	-72	46	4.06	104
	Middle cingulate cortex	L	23	-4	-28	32	5.04	72
	Inferior parietal cortex	R	40	58	-32	38	4.37	66
	Orbitofrontal cortex	R	11	30	30	-16	4.54	56
	Cerebellum	L		-10	-70	-24	4.06	51
aff > cog	Inferior parietal cortex extending to TPJ	R	40	62	-34	38	4.94	292
	SMA	L	6	-16	0	66	5.74	208
	Middle cingulate cortex	R/L	24/23	2	-14	34	4.43	105
	Anterior cingulate cortex	R	24	2	22	28	3.85	63
	Precentral gyrus extending to somatosensory cortex	L	4/6/1	-34	-30	64	3.99	47

*Note*. Localisation of significant brain activation, coordinates drawn from local maxima. Height threshold *T* = 3.19. Extend threshold *k* = 46 voxels. aff = affective theory of mind condition. cog = cognitive theory of mind condition. L = left. phy = control condition. R = right. SMA = supplementary motor area. STS = superior temporal sulcus. TPJ = temporoparietal junction.
